# Effect of orientation on polarization switching and fatigue of Bi_3.15_Nd_0.85_Ti_2.99_Mn_0.01_O_12_ thin films at both low and elevated temperatures

**DOI:** 10.1186/s11671-019-2949-3

**Published:** 2019-04-02

**Authors:** Wanli Zhang, Yanhu Mao, Shaoan Yan, Minghua Tang, Yongguang Xiao, Suihu Dang, Wenxi Zhao, Guangzhao Wang

**Affiliations:** 1 School of Electronic Information Engineering, Yangtze Normal University, Chongqing, 408100 China; 2 Key Laboratory of Welding Robot and Application Technology of Hunan Province, School of Mechanical Engineering, Xiangtan University, Xiangtan, 411105 China; 3 Engineering Research Center of Complex Tracks Processing Technology and Equipment of Ministry of Education, School of Mechanical Engineering, Xiangtan University, Xiangtan, 411105 China; 4 Key Laboratory of Key Film Materials & Application for Equipments (Hunan Province), School of Material Sciences and Engineering, Xiangtan University, Xiangtan, 411105 Hunan China; 5 Hunan Provincial Key Laboratory of Thin Film Materials and Devices, School of Material Sciences and Engineering, Xiangtan University, Xiangtan, 411105 Hunan China

**Keywords:** Polarization switching, BNTM, Domain pinning, Temperature-dependent fatigue

## Abstract

Bi_3.15_Nd_0.85_Ti_2.99_Mn_0.01_O_12_ (BNTM) thin films with (200)-orientations, (117)-orientations, and mixed-orientations were prepared by sol-gel methods. The influence of orientations on polarization fatigue behaviors of BNTM thin films were systematically investigated at both low and elevated temperatures. It was found that the changed trends of the polarization fatigue of (200)-oriented and (117)-oriented BNTM thin films at elevated temperatures were opposite. The fatigue properties become exacerbated for the (200)-oriented ones and become improved for the (117)-oriented ones, while the reduction of remanent polarization first decreases and then increases for the mixed-oriented ones. It can be assumed that the different roles played by domain walls and interface layer with increasing *T* in these thin films have caused such differences, which was certified by the lower activation energies (0.12–0.13 eV) of (200)-oriented BNTM thin films compared to those of BNTM thin films (0.17–0.31 eV) with other orientations through the temperature-dependent impedance spectra analysis. With the aid of piezoresponse force microscopy (PFM), the non-neutral tail-to-tail or head-to-head polarization configurations with greater probabilities for (117)-oriented and mixed-oriented thin films were found, while a majority of the neutral head-to-tail polarization configurations can be observed for (200)-oriented ones.

## Background

Bi_4_Ti_3_O_12_ (BIT)-based layered ferroelectric thin films have always been one of the most potential ferroelectric materials to replace the commercial (Pb, Zr)TiO_3_ (PZT)-based ferroelectric random access memory (FRAM) for its high curie temperature, large remanent polarization, and good anti-fatigue properties [1–3]. The lattice constants of BIT crystal along the *c*-axis, *a*-axis, and *b*-axis were 3.284 nm, 0.544 nm, and 0.541 nm at 300 K, respectively. BIT thin films also show anisotropic polarizations, which are about 4 and 50 μC/cm^2^ along its *c*- and *a*-axis, respectively [4]. There are numerous factors such as layer thickness, precursor solution, and annealing condition that affect the orientation of Nd-substituted BIT (Bi_3.15_Nd_0.85_Ti_3_O_12_, BNT) films [5–7]. Hu discovered that different thicknesses of each spin-on coating layer can favor BNT films with different orientations [5]. Yu et al. proposed that 0.10 M precursor solution for BNT showed the best ferroelectric and dielectric properties [6]. Zhong et al. reported that Bi_3.15_Nd_0.85_Ti_2.99_Mn_0.01_O_12_ (BNTM) thin film with an annealing temperature of 750 ^o^C showed higher tunability and dielectric constant than BNT thin film annealing under a temperature of 700 °C [7]. But high leakage current and poor fatigue properties can be caused by the evaporation of bismuth under high annealing temperatures. Moreover, it has also reported that BNT thin films with different orientations exhibit varying polarization fatigue behaviors [8]. However, the reason for why different orientations showed varying fatigue characteristics at elevated temperatures was still not very well understood.

Ferroelectric-based memories may operate in temperature range from − 40 to 125 °C, which can be elusive to understand the temperature-dependent change of fatigue behaviors of ferroelectric materials. It has been reported that the fatigue endurance for BNT thin films showed improved fatigue resistance from 25 to 125 °C, which can be attributed to the fact that the effect of domain unpinning enhanced more rapidly with increasing temperature than that of domain pinning. [9]. However, an opposite fatigue behavior has been observed in Bi_3*.*25_Sm_0*.*75_V_0*.*02_Ti_0*.*98_O_12_ thin films, where fatigue resistance deteriorates with increasing temperatures [10]. It can be elucidated that lots of affecting factors get together to decide the trend of fatigue behaviors at elevated temperatures as reported in our previous work [11]. Zhang et al. have studied the polarization switching properties of BNT thin films at elevated temperatures and concluded that the enhanced effect of electron injection can produce more highly mobile defect charges due to a lower Schottky barrier at high temperatures compared to that at low temperatures, which can induce pinned domain walls and serious fatigue [12]. However, earlier reports mainly studied on macroscopic performance tests and neglected microscopic domain dynamics which are considered to mainly affect the polarization switching and fatigue behaviors. With the aid of impedance spectra techniques, PFM and first-principles theory, the microscopic domain evolution and activation energies of oxygen vacancies of BiFeO_3_ thin films can be successfully observed during the polarization fatigue tests [13]. Thus, the studies of microscopic domain dynamic and transport law of oxygen vacancies will be helpful to further understand the fatigue behaviors of anisotropic BNTM thin films at an elevated temperature (*T*).

In the following section, polarization switching and fatigue properties of BNTM thin films with (200)-orientations, (117)-orientations, and mixed-orientations were studied at elevated temperatures from 200 to 475 K. The temperature-dependent fatigue behaviors of these thin films were also illuminated. The combination of temperature-dependent impedance spectra and PFM tests was made to learn the transport mechanisms of oxygen vacancies and microscopic evolution of domains. Different transport mechanisms of carriers of BNTM thin films with varying orientations for fatigue behaviors at elevated *T* will be discussed in detail.

## Methods

All chemicals and reagents were supplied by Sinopharm Chemical Regent, Co., Ltd. The starting precursor materials were Bi(NO_3_)_3_·5H_2_O (purity ≥ 99.0%), Nd(NO_3_)_3_·6H_2_O (purity ≥ 99.0%), Ti(OC_4_H_9_)_4_ (purity ≥ 99.0%) and Mn(CH_3_COO)_2_·4H_2_O (purity ≥ 99.0%). The solvents were 2-methoxyethanol (purity ≥ 99.0%) and glacial acetic acid (purity ≥ 99.5%) with acetyl acetone (purity ≥ 99.0%) as a chelating agent. Ten percent excess of bismuth nitrate was added to compensate for possible bismuth loss during the high-temperature process. The precursor solutions were adjusted to 0.04 M, 0.08 M, and 0.1 M, which correspond to BNTM-1, BNTM-2, and BNTM-3 thin films, respectively. These detail works can be found in our previous studies [14, 15]. The spin-on films were repeated ten times at 700 °C for 2.5 min in O_2_ for BNTM-1 and were repeated four times at 700 ^o^C for 5 min in O_2_ for BNTM-3, while the annealing processes were repeated for four times at 650 ^o^C for 2.5 min in O_2_, and the final layer was given an extra thermal process at 720 °C for 5 min in O_2_ for BNTM-2. Pt top electrodes were deposited with a diameter of 200 μm through DC sputtering.

X-ray diffraction (XRD) with Cu-*K*ɑ radiation was used to study texturing state and crystallographic structure of such thin films. Scanning electron microscope (SEM, Japan, Hitachi S4800) was conducted to characterize the surface and cross-sectional morphologies of these films. Semiconductor device analyzer (Agilent, USA, B1500A) which was combined with a temperature-controlled probe system was used to measure temperature-dependent dielectric properties and AC impedance spectra of such films. A commercially available *Z*-view software was used to analyze the impedance results. Ferroelectric test systems (USA, Radiant Technologies Precisions workstations) were used to measure the polarization fatigue properties. PFM (piezoresponse force microscopy) tests were conducted by using AFM (atomic force microscopy) system (MFP-3D, USA, Asylum Research) under an ambient condition. A platinum-coated silicon cantilever (radius 15 nm, spring constant 2 N/m) was used to scan with a tip lift height of 30 nm at 35 kHz.

## Results and Discussion

**Fig. 1.**
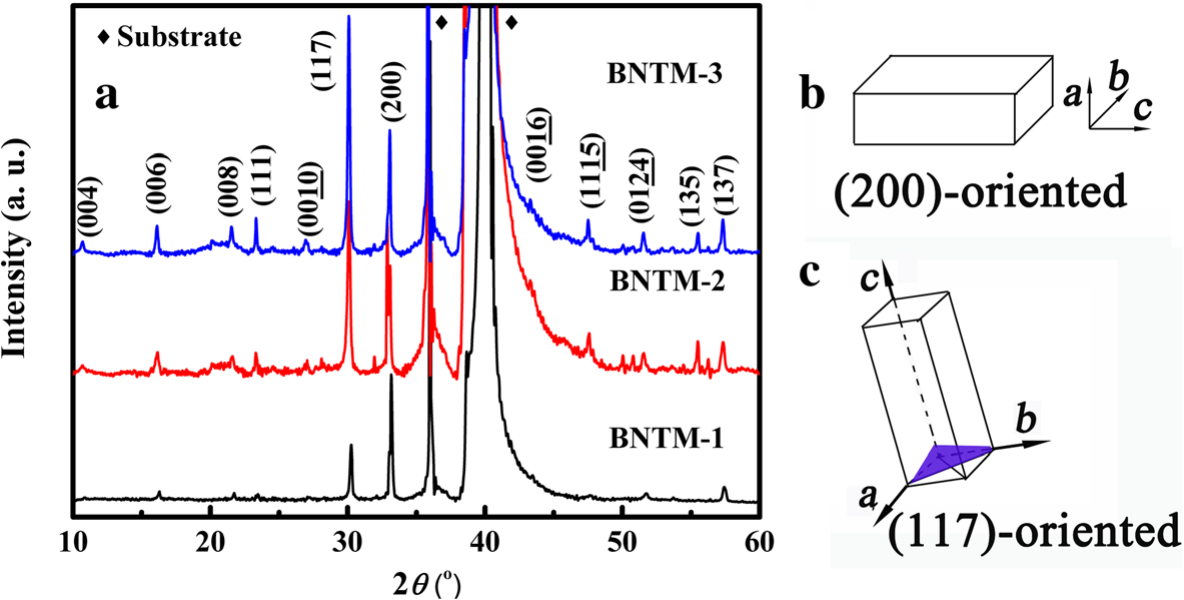
XRD patterns of BNTM-1, BNTM-2, and BNTM-3 thin films (**a**) and schematic diagram of (200)-grain growth (**b**) and (117)-grain growth of thin films (**c**)

**Fig. 2.**
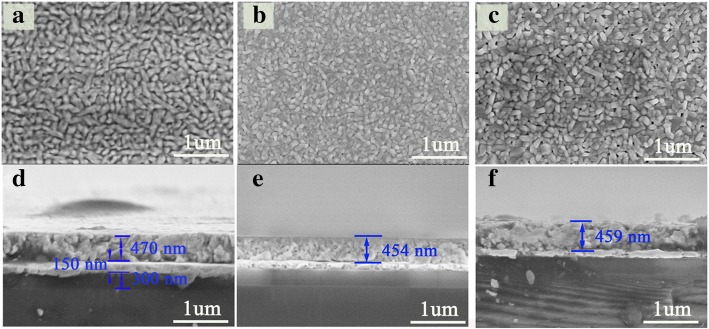
SEM surface and cross-section images: **a**, **d** BNTM-1; **b**, **e** for BNTM-2; **c**, **f** for BNTM-3

XRD patterns of BNTM-1, BNTM-2, and BNTM-3 thin films were shown in Fig. 1. To quantify the texturing state, the degrees of orientation are defined as *α*_hkl_ = *I*_(hkl)_/(*I*_(006)_ + *I*_(117)_ + *I*_(200)_), where *I*_(hkl)_ is the XRD peak intensity of (hkl) crystal plane. The degrees of *α*_200_ and *α*_117_ of BNTM-1, BNTM-2, and BNTM-3 thin films were found to be 63.50% and 29.23%, 43.22% and 48.5%, and 32.11% and 60.2%, respectively. A (200)-oriented growth of BNTM-1 and (117)-oriented growth of BNTM-3 were observed, while a mixed-preferred growth was presented in BNTM-2. The surface and cross-section of such thin films are observed through SEM methods as shown in Fig. 2a–g. The surface of BNTM-1, BNTM-2, and BNTM-3 thin films is mainly composed of bullet-shaped grains, a mixture of plate-like grains, and rod-like grains through the observation in Fig. 2a–c, respectively, which was also reported in others’ works [16]. Film thicknesses of BNTM-1, BNTM-2, and BNTM-3 were estimated to be 470 nm, 454 nm, and 459 nm through the cross-sectional SEM images (as shown in Fig. 2d–g), respectively. As mentioned above, layer-by-layer crystallization was adopted in the preparation of BNTM thin films. The growth of (117)-oriented crystals was favored by the thicker spin-coating layer, while the growth of (200)-oriented crystals was not restricted by the layer thickness owing to the geometrical effect as shown in Fig. 1b and c. The thicknesses of each spin-coating layer of BNTM-1, BNTM-2, and BNTM-3 thin films were estimated to be 47 nm, 91 nm, and 115 nm, respectively, which favor the (200)-oriented, the mixed-oriented, and the (117)-oriented BNTM thin films. These results have also been reported by Hu and Wu [5, 17].

**Fig. 3.**
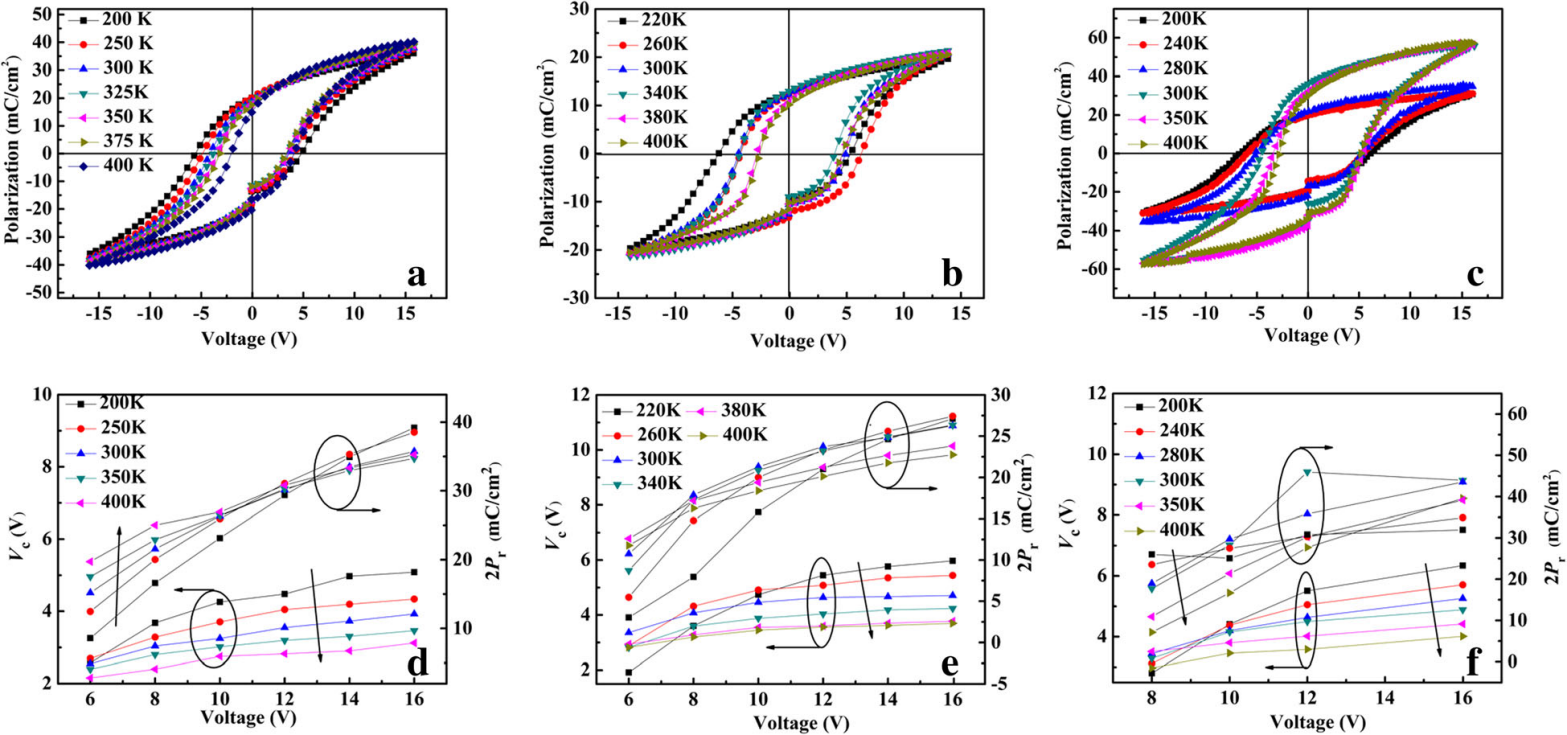
*P*-*V* hysteresis loops measured with the *V*_m_ of 16 V at 1 kHz and plots of *V*_c_ and 2*P*_r_ as functions of *V*_m_ at elevated temperatures: **a**, **d** for BNTM-1; **b**, **e** for BNTM-2; **c**, **f** for BNTM-3

The *P-V* hysteresis loops of BNTM-1, BNTM-2, and BNTM-3 thin films from 200 to 400 K measured with the maximum voltage (*V*_m_) of 16 V were exhibited in Fig. 3a–c. The remanent polarization 2*P*_r_ and coercive voltage 2*V*_c_ of such films strongly depend on *T* as shown in Fig. 3d–f, where the average coercive voltage *V*_c_ (*V*_c_ = (*V*_c_^+^-*V*_c_^-^)/2) and 2*P*_r_ as a function of *T* under different *V*_m_. It can be concluded that 2*P*_r_ of BNTM-1 first increases as *V*_m_ is less than 10 V, and decreases when *V*_m_ is more than 10 V with increasing *T*, while 2*P*_r_ of BNTM-2 and BNTM-3 always first increases from 220 to 300 K and then decreases from 300 to 400 K under the whole range of *V*_m_. It can be explained by the larger depolarization field at film/electrode interfaces of BNTM-2 and BNTM-3 which is caused by the higher density of domain walls, while its amounts at interfaces are lower for BNTM-1. The values of *V*_c_ of BNTM-1 decrease with increasing *T* as the values of *V*_m_ increase from 6 to 16 V, while its values of BNTM-2 and BNTM-3 first increase and then decrease with increasing *T* under the values of *V*_m_ from 8 to 10 V. It should be triggered by the competition of the nucleation rate of domains and domain pinning-unpinning with increasing *T*, where the nucleation rate of domains (*n*) and the activation electric field (*α*) can be expressed as*n* ∝ exp(−*α*/*E*). Thus, *n* plays a decisive role to determine the values of *V*_c_ at low *T* and small *V*_m_, and an increasing *V*_c_ will be increased with a higher nucleation rate of domains. The domain wall velocity has strongly determined the probability of domain wall pinning after reaching the saturation point of nucleation rate of domains at high *V*_m_ and *T*. Domain wall velocity (*v*) and the energy barrier for domain growth (*U*_0_) can be expressed as *ν* ∝ exp(−*U*_0_/*k*_*B*_*T*), where *k*_B_ means the Boltzmann constant [18]. With the increasing *T*, the domain unpinning effect has been strongly enhanced by the increasing *v*. Thus the fact that *V*_*c*_ decreases with the increasing *T* at the saturation value of *V*_m_ can be due to the higher *v*.

**Fig. 4.**
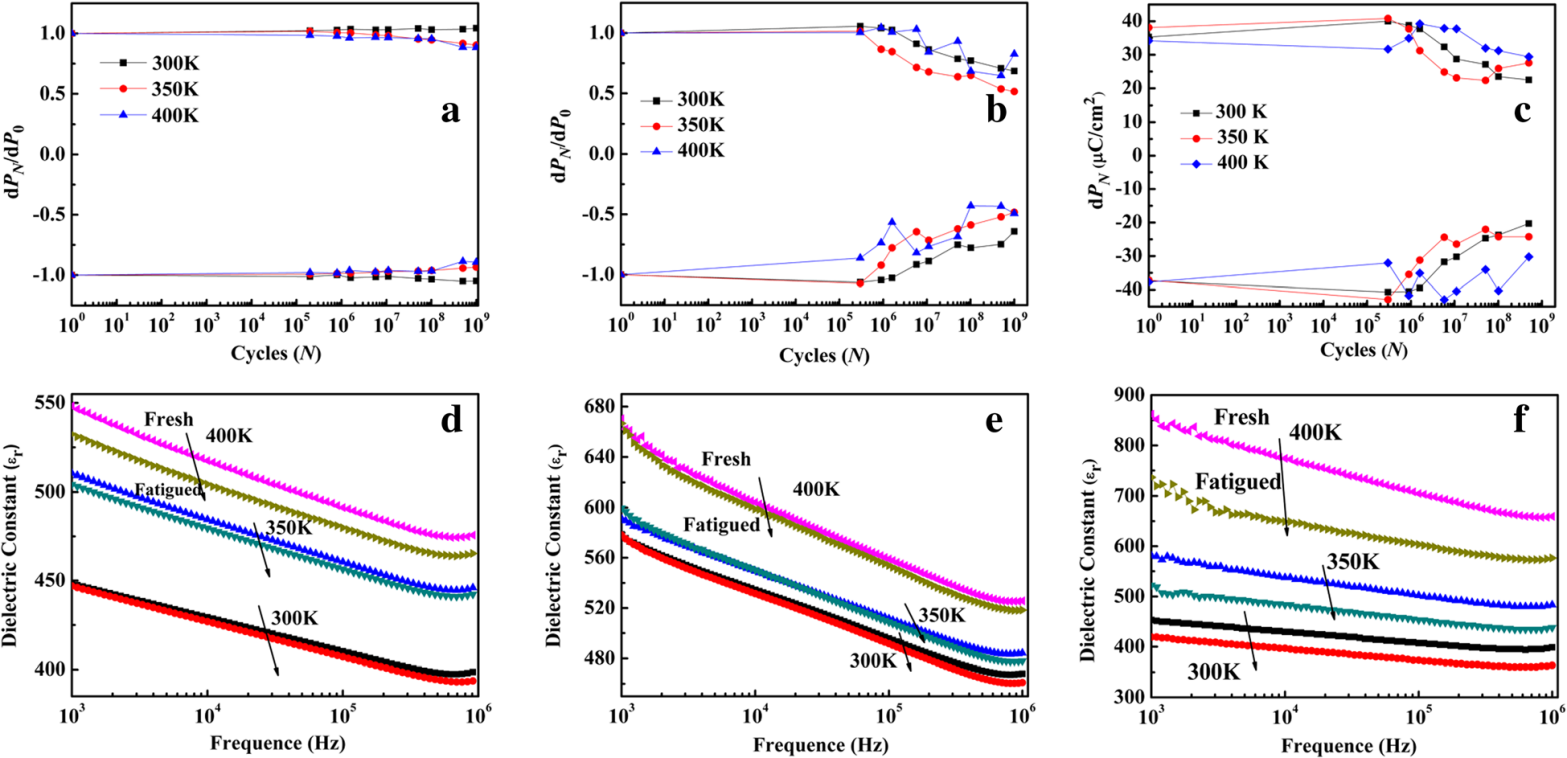
Plots of polarization fatigue curves and dielectric constant (*ε*_r_) vs frequency at both fresh and fatigued condition: **a**, **d** for BNTM-1; **b**, **e** for BNTM-2; **c**, **f** for BNTM-3

The fatigue characteristics of BNTM-1, BNTM-2, and BNTM-3 from 300 to 400 K were displayed in Fig. 4a–c. The pulse amplitudes were 10 V and 8 V for the reading and fatigue process, respectively. The relationship of $\pm {dP}_N={\left(\pm {P}_r^{\ast}\right)}_N-{\left(\pm {P}_r^{\wedge}\right)}_N$ can be described that *N* is the number of switching cycles,*P*_*N*_ is the total polarization, ${P}_r^{\ast }$ is the switched remanent polarization between the two opposite polarity pulses, and ${P}_r^{\wedge }$ is the non-switched remanent polarization between the same two polarity pulses. After 1 × 10^9^ cycles pulse switching, the reductions of d*P*_*N*_ *of* BNTM-1, BNTM-2, and BNTM-3 were 0%, 32.5%, and 41.2% at 300 K, 7.4%, 51.4%, and 31.2% at 350 K, and 11.3%, 34.5%, and 15.7% at 400 K, respectively. The fatigue characteristics of BNTM-1 become more serious and these of BNTM-3 show a reverse trend from 300 to 400 K, while the fatigue characteristics of BNTM-2 become more serious from 300 to 350 K, and get improved from 350 to 400 K. At first*,* the improved fatigue properties of BNTM-3 from 300 to 400 K should be due to the enhanced effect of domain wall unpinning [11, 18–20]. It can be consumed that the competition between domain pinning and the growth of dead layer has always been an obvious effect on polarization fatigue [21, 22]. As for BNTM-1, the growth of the dead layer is the dominant factor, and the long-range diffusion of oxygen vacancies becomes enhanced with increasing *T* and contributes to the increase of the thickness of the dead layer, which can be also certified by the decrease of dielectric response after fatigue process from Fig. 4d. As for BNTM-2, the effect of dead layer growth first plays a major role with *T* from 300 to 350 K during the fatigue tests, and then the enhanced domain unpinning effect leads to improved fatigue properties from 350 to 400 K. It was also discussed in some other works [22, 23].

The plots of dielectric constant (*ε*_r_) vs frequency before and after the fatigue process were further conducted to investigate the dead layer growing effect as shown in Fig. 4d–f. The values of *ε*_r_ of such thin films increase with increasing *T*, which indicates that the domain unpinning effect becomes stronger with the increasing *T.* The change in the values of *ε*_r_ of BNTM-1 and BNTM-3 after fatigue process increases with increasing *T*. It can be explained by the combined effect of long-range diffusion of removable carriers and the dead layer’s growth at elevated *T*. As for BNTM-1 and BNTM-3, the thickness of the dead layer increases with increasing *T* and becomes the main influence on the value of *ε*_r_, which leads to the reduction of *ε*_r_ of BNTM-1 and BNTM-3. However, the change in the *ε*_r_ of BNTM-2 showed a weak correlation, which was explained that a large amount of charged domain wall formed by oxygen vacancies’ migration during the fatigue process had taken part in the dielectric response, which caused the increase of *ε*_r_ for BNTM-2.

**Fig. 5.**
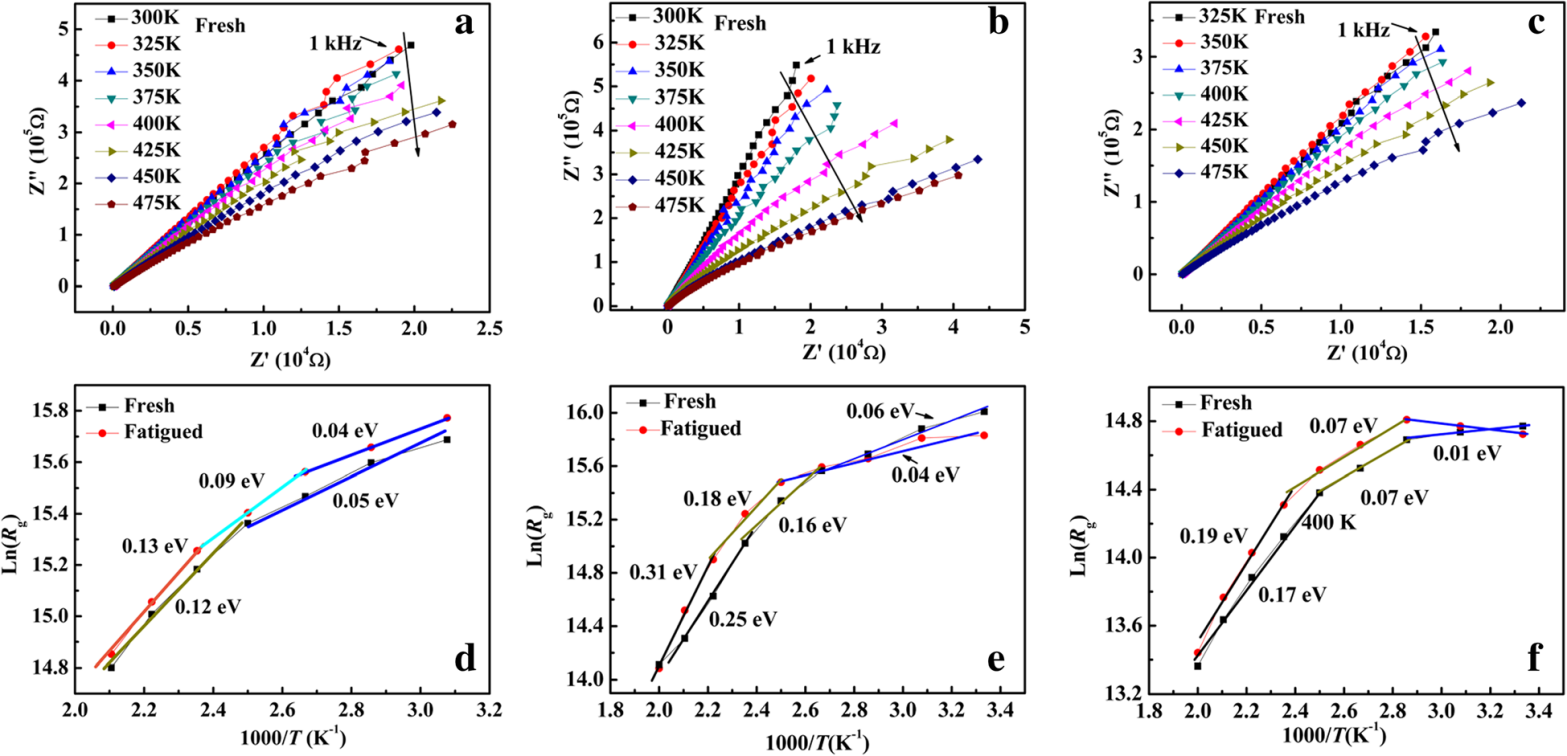
Impedance diagrams at elevated temperature and Ln(*R*_g_) vs 1000/*T* Arrhenius plots at both fresh and fatigued condition: **a**, **d** for BNTM-1; **b**, **e** for BNTM-2; **c**, **f** for BNTM-3

**Fig. 6.**
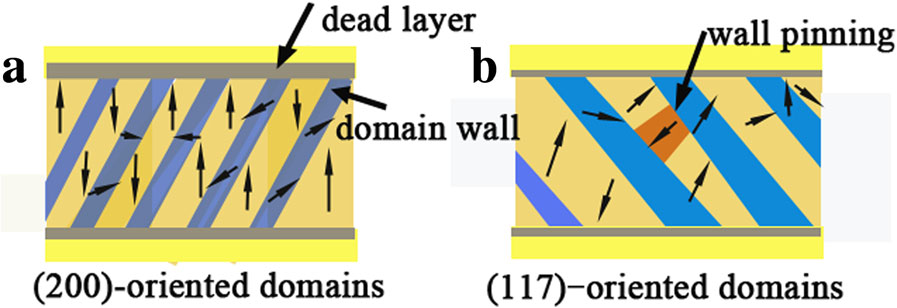
**a**, **b** Schematic domain structure in the *a*–*b* plane of the (200)-oriented and (117)-oriented BNTM thin films (the domain orientation can trace the arrows)

AC impedance spectra tests were used to study the conductance mechanism before and after the fatigue process with the temperature range from 300 to 475 K. Fig. 5a–c shows the real and imaginary impedance (*Z´* and *Z"*) as frequency decreases from 1 MHz to 1 kHz. The grain contribution can be reflected by high-frequency arcs. The nonlinear least square fitting were conducted to estimate resistances of grains (*R*_g_) of BNTM films, which was also reported by Bai et al. [24]. The *R*_g_ follows Arrhenius’ relationship as*R*_*g*_ ∝ exp(−*E*_*a*_/*k*_*B*_*T*), where *E*_a_ represents average activation energy of carriers during conductance process and *k*_B_ means Boltzmann’s constant [25]. The curves of ln(*R*_g_) vs 1000/*T* were shown in Fig. 5d–f. It has been found that the value of *R*_g_ increases a little after 1.6 × 10^9^ pulse cycles, which can be elucidated that the population of carriers increased with increasing *T* and a part of oxygen vacancies or injected electrons was trapped by charged domain walls during the fatigue process [26, 27]. The values of *E*_a_ for BNTM-1 were 0.12-0.13 eV from 425 to 475 K and much smaller than the values of BNTM-2 and BNTM-3. The large values of *E*_a_ (0.12-0.31 eV) are generally considered as the contribution of the migration of oxygen vacancies within their clusters [25]. It can be estimated that long-range diffusion of oxygen vacancies happens more easily in BNTM-1 thin film, which was further accounted for that the density of domain walls of (200)-oriented thin films was less than that of (117)-oriented and mixed-oriented thin films. The schematics of domains and the domain walls of (200)-oriented and (117)-oriented BNTM thin films were made as shown in Fig. 6a–b. It can be seen that the (200)-oriented thin films mainly consist of 180°-domain and, the width of domain wall is much smaller than that of (117)-oriented domains, which have strong horizontal component of polarization. The tail-to-tail or head-to-head polarization configurations which can induce the pinning effect for domain walls can happen more easily with (117)-oriented domains. Thus the question why (200)-oriented BNTM thin films show the opposite fatigue behaviors with increasing *T* compared to that in (117)-oriented BNTM thin films can be explained. For BNTM-1 mainly consisted of (200)-oriented domains, the diffusion of oxygen vacancy should be a determined role for the fatigue behaviors with increasing *T*. And for BNTM-3 with a majority of (117)-oriented domains, domain walls with larger width which depend on the temperature should be the main cause. The intense diffusion of oxygen vacancies with increasing *T* can facilitate the growth of a dead layer which makes serious fatigue, while the width of domain wall can be smaller with increasing *T.* Thus an improved fatigue properties can achieve.

**Fig. 7.**
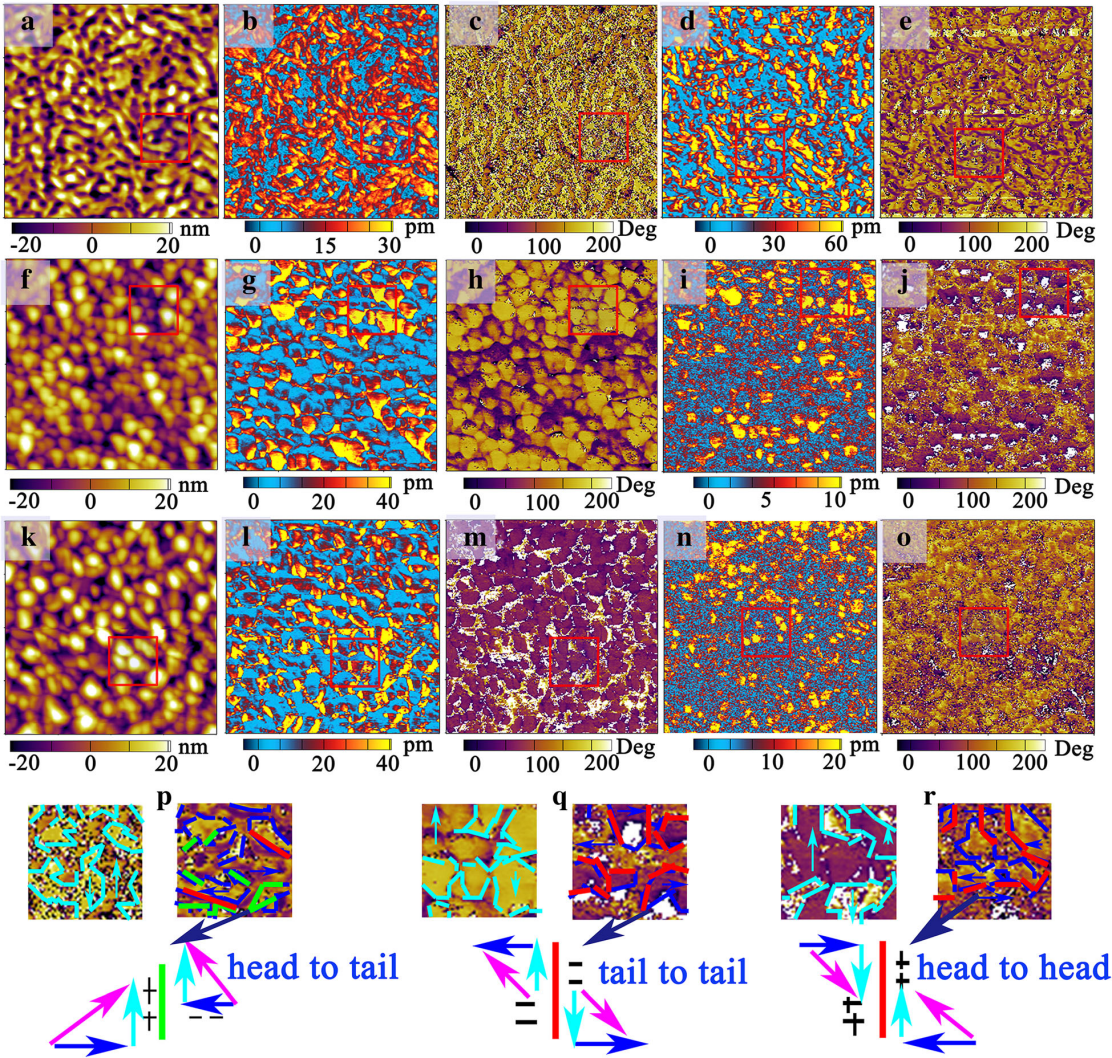
AFM surface topography, OP PFM amplitude images, OP PFM phase images, IP PFM amplitude images, IP PFM phase images, and zoomed-in PFM images of a specific region in the red solid square: **a**–**e, p** for BNTM-1, **f**–**j, q** for BNTM-2, **k**–**o**, **r** for BNTM-3, respectively, and the scanning area is 2 × 2 μm^2^

In order to verify our correctness of aforementioned models, the microscopic domain structures of BNTM-1, BNTM-2, and BNTM-3 thin films were studied through the PFM method. AFM surface topography, OP (out-plane) PFM amplitude images, OP PFM phase images, IP (in-plane) PFM amplitude images, IP PFM phase images, and zoomed-in PFM images of a specific region in the red solid square of such films were shown in Fig. 7a–o. The regions with bright yellow and dark colors in OP phase images correspond to vertically up or down 180°-domains, while the regions with rich yellow and dark colors in IP image correspond to laterally left or right 90°-domains. It can be seen that the phases of laterally right or left 90°-domains are more obvious for BNTM-2 and BNTM-3 than those of BNTM-1 as shown in Fig. 7p–r, which has further elucidated that (117)-oriented domains have a strong horizontal component of polarization. IP PFM images of the zoomed-in specific region with red solid squares were shown in Fig. 7p–r. The cyan dotted lines correspond to the boundaries of vertically up and down 180°-domains in OP phase images as shown in Fig. 7p–r, while the blue dotted lines correspond to the boundaries of laterally left and right 90°-domains in IP images. When cyan dotted lines are just located at the boundaries of dark and bright regions in IP phase images which are marked with blue dotted lines, the polarization configurations with tail-to-tail or head-to-head structures which were marked with red dotted lines in Fig. 7p–r will be formed and lead to the accumulation of opposite charge for domain walls. It can be concluded that the non-neutral tail-to-tail or head-to-head polarization configurations can happen with greater probabilities for BNTM-2 and BNTM-3 thin films compared to those for BNTM-1 thin films as shown in Fig. 7p–r. Therefore, the density of pinned domain walls and width of domain wall have determined the temperature-dependent fatigue behaviors for (117)-oriented thin films. Thus domain walls with higher velocity and less possibility to capture oxygen vacancies can realize the improved fatigue at elevated temperatures as compared to those at lower temperatures [28].

## Conclusions

In conclusion, the mechanisms of orientations on temperature-dependent polarization switching and fatigue properties of BNTM thin films were systematically expounded. It was found that the fatigue properties become exacerbated for (200)-oriented thin films and become improved for (117)-oriented ones with increasing *T*. The accumulation of oxygen vacancies at the interface and domain walls with larger width should be the determined roles for the fatigue behaviors of (200)-oriented and (117)-oriented thin films with increasing *T*, respectively. The intense diffusion of oxygen vacancies with increasing *T* can facilitate the growth of a dead layer which makes serious fatigue, while the width of domain wall becomes smaller and brings improved fatigue properties affected by an enhanced domain unpinning effect with increasing *T*. A lower activate energy of 0.12–0.13 eV was found for (200)-oriented BNTM thin films as compared to those of 0.17-0.19 eV for (117)-oriented ones. The non-neutral tail-to-tail polarization configurations with greater probabilities for (117)-oriented and mixed-oriented thin films were found, while a majority of the neutral head-to-tail polarization configurations can be observed for (200)-oriented ones. Therefore, the intense diffusion of oxygen vacancies and the properties of domain walls have determined the differences of temperature-dependent fatigue behaviors of BNTM thin films with different orientations.

## Abbreviations

AFM: Atomic force microscopy
BIT: Bi_4_Ti_3_O_12_
BNT: Nd-substituted BIT
BNTM: Bi_3.15_Nd_0.85_Ti_2.99_Mn_0.01_O_12_
*E*_a_: Average activation energy of carriers
FRAM: Ferroelectric random-access memory
IP: In plane
*k*_B_: Boltzmann constant
*n*: Nucleation rate of domains
OP: Out plane
PFM: Piezoresponse force microscopy
*P*_N_: Total polarization
*P*_r_: Remanent polarization
*P*_r_^*^: Switched remanent polarization
*P*_r_^^^: Non-switched remanent polarization
PZT: (Pb, Zr)TiO_3_
*R*_g_: Resistances of grains
SEM: Scanning electron microscope
*U*_0_: Energy barrier for domain growth
*V*_c_: Coercive voltage
*V*_m_: Maximum voltage
XRD: X-ray diffraction
*Z*”: Imaginary impedance
*Z*': Real impedance
*α*: Activation electric field
*ε*_r_: Dielectric constant
